# Editorial: “Improving impacts of mental health treatment on substance misuse and vice versa”

**DOI:** 10.3389/frcha.2026.1816035

**Published:** 2026-04-22

**Authors:** Ty A. Ridenour, Chardée A. Galán, Caroline Oppenheimer

**Affiliations:** 1 Department of Psychiatry, University of North Carolina, Chapel Hill, NC, United States; 2 School of Pharmacy, University of Pittsburgh, Pittsburgh, PA, United States; 3 Department of Psychology, The Pennsylvania State University, State College, PA, United States; 4 RTI International, Research Triangle Park, NC, United States

**Keywords:** adolescence, editorial, mental health, prevention, substance & alcohol use, treatment

Recent epidemics of harmful substance use (SU) span the globe including psychostimulants in Europe and the Middle East, opioids in North America and Asia, and inhalants in South and Central America. These trends have been accompanied by parallel epidemics in mental health disorders, drug overdoses, and broader societal burdens such as crime or acute and chronic medical illness (1, 2). Adolescents and young adults have been disproportionately impacted by both SU and its public health challenges (3, 4). Understanding how mental health problems intersect with SU etiology, and applying this insight to prevention and treatment efforts, has the potential to improve outcomes for millions of individuals, their families, and their communities.

A substantial body of theory and empirical work delineates developmental cascades from mental health difficulties to SU (5, 6). Conversely, it is well-established that SU and SU disorders increase risk for subsequent psychological problems (7–9). Despite these insights, comparatively less progress has been made in translating this knowledge to improve prevention and healthcare for youth at elevated risk for SU and/or mental illness (10).

Building on a well-established literature, this Research topic highlights new work that refines our understanding of how substance use and mental health challenges co-occur and unfold across development. The included studies span etiology, screening, prevention, intervention, and healthcare engagement.

Ramakrishnan and colleagues examined vulnerability to mental health challenges and SU among children with a family history of SU using data from the Adolescent Brain Cognitive Development study (youth ages 9–10). Compared to children without such a history, children with a family history of SU showed greater internalizing and externalizing symptoms, greater impulsivity, and more errors in reward prediction. Specifically in children with a family history of harmful SU, five subgroups were derived statistically based on sociodemographic characteristics. These subgroups differed in their types and average level of impulsivity as well as errors in predicting rewards, demonstrating that by age 10 childhood vulnerabilities to mental health difficulties and SU are already detectable. These findings reinforce the importance of early detection and profiling of a youth's vulnerabilities to SU and mental health problems to facilitate selective or indicated prevention.

Barry and colleagues investigated whether anxiety or depression predicted later SU among high school students residing on or near a Tribal reservation in the U.S. Great Plains. Students’ anxiety and depressive symptoms prospectively predicted increased use of alcohol, cannabis and/or prescription opioids a few months later. Their findings suggest that internalizing symptoms may function as a precursor to SU and its consequential problematic sequelae.

Hails and colleagues investigated impact of the well-researched Family Check-Up prevention program (designed to enhance parenting skills to prevent their offspring's substance use and improve mental health) on the recipient parents' anxiety levels. They conducted a randomized controlled trial of an online version of the Family Check-Up program with 356 parents who had a lifetime and/or current history of harmful SU or elevated depressive symptoms, and a child between ages 1.5–5 years The authors found parents' adolescent-onset cannabis use, independent of current use, was associated with parents' higher levels of anxiety and depressive symptoms in adulthood. Further, adolescent-onset use moderated intervention effects, such that the FCU was particularly effective at reducing anxiety symptoms among those wit a history of early cannabis use. These findings corroborate other clinical trials demonstrating broader benefits of the Family Check-Up for both parents and their children, including improved mental health and reduced substance use (11–13).

The Youth Risk Index has been used in prior work to identify youth at elevated risk for substance use and conduct problems, including a randomized controlled trial of the Family Check-Up in a primary care setting where it was used to detect youth who would benefit from prevention services (14). In this Research Topic, Ridenour and colleagues evaluated psychometric properties of the Youth Risk Index. Their analyses indicated that the Youth Risk Index demonstrates excellent concurrent and predictive validity for SU and conduct problems in both clinical and community samples. Youth Risk Index scores were associated with a broad range of risk factors including personal and neurocognitive characteristics, family and school environments, and depression. Factor analyses suggested that Youth Risk Index items are comprised of three subscales, disinhibition, peer conduct problems, and social contagion, reflecting foundational sources of liability to both mental health difficulties and multiple health risk behaviors.

Even after prominent etiological factors are identified, and efficacious screening protocols and prevention programs are developed, barriers to care can limit their population impact (10). Chen et al. addressed this challenge by evaluating strategies to encourage engagement and retention in treatment among adolescents of historically marginalized backgrounds who were receiving SU treatment. They reported that providing meal incentives and transportation services to attend sessions was effective in promoting engagement and retention. These findings illustrate a core principle of implementation science: addressing multiple, context-specific barriers is essential to achieving reach and real-world effectiveness.

Overall, this Research Topic coincides with growing recognition that effective intervention for co-occurring mental health challenges and harmful SU must be grounded in their shared and interacting etiologies. The U.S. National Institute of Mental Health Research Domain Criteria (RDoC) framework was developed in part to address “an emerging concern that the current diagnostic system was hampering translational research” (15). By emphasizing cross-diagnostic mechanisms across six broad domains of human functioning on mental health and integrating behavioral and neurobiological levels of analysis, RDoC provides a structure for linking etiology to treatment targets. The etiologically informed screening tools and preventive interventions highlighted in this collection reflect this translational vision.

Important areas of needed research that this research topic did not address are learning how cultural mores and societal factors contribute to mental health development, SU etiology, and treatment and prevention of potentially poor outcomes (16, 17). As noted during our introductory text, SU disorder and related problems are prominent issues globally; however, even the substances of choice and which drugs are considered acceptable to consume differ widely among regions of the world. Similiarly diverse are diverse are cultural norms regarding emotional expression, shame and stigma, etiology of mental health and illness, discriminatory stereotypes, and coping strategies (17). Barriers and access to treatment, capacity for internet purchase of potent and potentially fatal substances, and quality of health information also vary across cultures and regions, further complicating the equifinality and multifinality of development related to SU and mental health as well as providing opportunities to improve developmental trajectories toward healthy and fulfilling outcomes.

In sum, four of the Research Topic's manuscripts illustrate the breadth of advances in evidence-based approaches to understanding etiology of adolescent mental health and risk health behavior and translating this knowledge into practice with their underpinnings aligning with the criteria within the RDoC. The fifth manuscript illustrates the importance of a new and rapidly growing scientific field, implementation science, to improve access to and delivery of mental health services and SU treatment. Continued integration of etiology research, screening, prevention, and implementation strategies offers great promise for reducing the global burden of adolescent mental health problems and SU.
